# Imaging of Morphological Background in Selected Functional and Inflammatory Gastrointestinal Diseases in fMRI

**DOI:** 10.3389/fpsyt.2020.00461

**Published:** 2020-05-20

**Authors:** Katarzyna Skrobisz, Grazyna Piotrowicz, Patrycja Naumczyk, Agnieszka Sabisz, Karolina Markiet, Grazyna Rydzewska, Edyta Szurowska

**Affiliations:** ^1^Department of Radiology, Medical University of Gdansk, Gdansk, Poland; ^2^Department of Gastroenterology, Self-Dependent Health Care Unit of Ministry of Interior, Gdansk, Poland; ^3^Institute of Psychology, University of Gdansk, Gdansk, Poland; ^4^II Department of Radiology, Medical University of Gdansk, Gdansk, Poland; ^5^Central Clinical Hospital of the Ministry of Interior, Warsaw, Poland

**Keywords:** functional magnetic resonance imaging (fMRI), irritable bowel syndrome (IBS), inflammatory bowel diseases, functional dyspepsia (fd), resting-state

## Abstract

The study focuses on evaluation of the Default Mode Network (DMN) activity in functional magnetic resonance imaging (fMRI) in resting state in patients with functional dyspepsia (FD) and irritable bowel syndrome (IBS), Crohn's disease and colitis ulcerosa (IBD) in comparison to healthy volunteers. We assume that etiology of both functional and non-specific inflammatory bowel diseases is correlated with disrupted structure of axonal connections. We would like to identify the network of neuronal connections responsible for presentation of symptoms in these diseases. 56 patients (functional dyspepsia, 18; Crohn's disease and colitis ulcerosa, 18; irritable bowel syndrome, 20) and 18 healthy volunteers underwent examination in MRI of the brain with assessment of brain morphology and central nervous system activity in functional imaging in resting state performed in 3T scanner. Compared to healthy controls' DMN in patients with non-specific digestive tract diseases comprised additional areas in superior frontal gyrus of left hemisphere, in left cingulum and in the left supplementary motor area. Discovered differences in the DMNs can be interpreted as altered processing of homeostatic stimuli. Our study group involved patients suffering from both functional and non-specific inflammatory bowel diseases. Nevertheless a spectrum of changes in the study group (superior frontal gyrus of the left hemisphere, in the left cingulum and in the left supplementary motor area) we were able to find common features, differentiating the whole study group from the healthy controls.

## Introduction

Functional disorders of the gastrointestinal tract (FGIDs) may explain from 25% up to 40% gastrointestinal tract (GI) derived symptoms in young adults (1). In recent years, an increase in incidence of non-specific inflammatory bowel diseases (IBDs), especially in people in their 20s to 40s, has been observed. Epidemiological data prove that chronic stress disorders underlie both FGIDs and IBDs (2, 3). Non-specific inflammatory bowel diseases, such as Crohn's disease or colitis ulcerosa, are characterized by chronic inflammation of GI tract. Stress seems not only to be a factor in exacerbation of symptoms in course of IBDs, but is considered an initiating agent as well (4).

Functional disorders of the gastrointestinal tract are diagnosed after exclusion of so called alert symptoms and structural diseases on bases of Rome III Criteria (5, 6). Etiology of FGIDs is elusive, pathogenesis theories include alteration in enteric motility, celiac hypersensitivity, intestinal barrier dysfunction, and underlying stress. FGIDs are often described as immunohormonal mucosal disorders or functional mucosal syndromes and symptoms seem to derive from a disturbance of balance between inflammatory cytokines released from intraepithelial lymphocytes and cytokines inhibiting inflammatory processes (7–9). There are ample data supporting theory that stress and other psychological disorders are factors in development of FIGDs. Patients present with increased levels of anxiety (fear), signs of depression (pessimism), and emotional tension (10–12). Moreover, the newest research in functional magnetic resonance imaging (fMRI) in resting state underline regional disturbances in brain activity in pathophysiology of functional dyspepsia. There is also extensive research into the role of disturbance of brain-gut-axis, which refers to bi-directional communication between the gut and the central nervous system, in etiology of functional gastrointestinal disorders (13, 14).

A number of studies contributed to the knowledge of neural underpinning of patients' sensations in gastric diseases and show two major findings. Abnormal interhemispheric interactions have been encountered in patients with FGIDs (15–17). The second finding focuses on mapping the brain regions—part of Default Mode Network (DMN)—connected to gastric sensations, including those involved in cognitive and emotional regulation (anterior cingulate cortex—ACC, prefrontal cortex—PFC, insula, temporal cortex, parahippocampal gyrus, and orbitofrontal cortex—OFC) (17–21) and associated with the “homeostatic afferent network” as described by Mayer Naliboff and Craig (22). Our study explored the common mechanisms underpinning different FGIDs. It is suggested that these disorders origin in central processing of the visceral stimuli (23–25). Patients with FGIDs and IBDs suffer from chronic visceral pain which is strongly connected with DMN. Studies reported that chronic pain is causing functional reorganization in the default mode network (26–28).

There is a limited number of studies which examined the role of DMN in inflammatory bowel diseases, among others, Liu et al. (29) reported disrupted local and global topological patterns of functional neural networks, including DMN in CD patients. Another study presented aberrantly activated regions in DMN in patients with ulcerative colitis (UC) (30).

We decided to focus on the default mode network as the one most activated during processing the self-specific stimuli (31).

## Materials and Methods

All patients gave written consent to participate in the study. Study has been approved by The Bioethical Committee of The Military Medical Council (Street Koszykowa 78, 00-909 Warsaw) (document 107/12 dated 22.06.2012).

### Functional Magnetic Resonance Imaging

Study group included patients with functional dyspepsia (FD), irritable bowel syndrome (IBS), and with non-specific inflammatory bowel diseases (IBDs) (Crohn's disease and ulcerative colitis)—FD, 18 (K, 13; M, 5; age range, 20–40 years; mean age, 33.28 years); IBS, 20 (K, 14; M, 6; age range, 23–44 years; mean age, 33.1 years); IBD, 18 (K, 10; M, 8; age range, 21–43 years; mean age, 26.83 years).

Patients suffering from FGIDs have been enrolled according to Rome III Criteria summarized in **Table 1** (5, 6). Patients with non-specific inflammatory bowel diseases have been qualified according to the anamnesis and results of additional tests, which included colonoscopy with histopathological assessment, gastrofiberoscopy, capsule endoscopy, and/or magnetic resonance enterography (MRE). To exclude stress associated with diagnosis a minimum period of three years from diagnosis has been established.

**Table 1:** Summary of Rome III Criteria on FGIDs.

	Diagnostic criteria*
1. Functional dyspepsia	Must include one or more of the following: –Bothersome postprandial fullness, –Early satiation, –Epigastric pain and/or burning. No evidence of structural disease (including at upper endoscopy) likely to explain the symptoms should be found.
1a. Epigastric pain syndrome (EPS)	Must include all of the following: –Pain or burning localized to the epigastrium of at least moderate severity, at least once a week, –Intermittent character of pain/burning, –Pain/burning should not be generalized or localized to other abdominal or chest regions, –Pain/burning should not be relieved by defecation or passage of ﬂatus. Criteria for gallbladder and sphincter of Oddi disorders should not be fulfilled.
1b. Postprandial distress syndrome (PDS)	Must include one or both of the following: –Bothersome postprandial fullness, occurring after ordinary-sized meals, at least several times a week, –Early satiation that prevents completing a regular meal, at least several times a week
2. Irritable bowel syndrome (IBS)	Recurrent abdominal pain or discomfort** at least 3 days/month in the last 3 months associated with two or more of the following: –Improvement with defecation, –Onset associated with a change in frequency of stool, –Onset associated with a change in form (appearance) of stool.

*Criteria fulﬁlled for the last 3 months with symptom onset at least 6 months prior to diagnosis.

**“Discomfort” stands for uncomfortable sensation, not described as pain.

The control group of 18 healthy volunteers consisted of nine women and nine men (age range, 24–47 years; mean age, 34.27 years).

The exclusion criteria comprised lack of fulfillment of Rome III Criteria for FGIDs, head trauma in anamnesis, severe additional diseases, depression, mental disorders, pregnancy, and/or lactation and well established contraindications to MRI.

Patients from both study and control group underwent a GAST questionnaire developed by one of the authors (GP). GAST questionnaire focuses on type of functional GI disease, anamnesis with emphasis on history of symptoms and concomitant diseases as well as sociodemographic data.

All patients subjected to research have been thoroughly introduced to its principles and given detailed information on examination procedures, especially on MR, also in order to reduce stress levels and avoid potential panic or claustrophobic attacks.

### Scanning Protocol

Functional and anatomical data sets were acquired in a 3T Achieva TX Scanner (Philips Healthcare, Best, the Netherlands) with the use of the eight-channel head coil. To evaluate brain morphology and exclude subjects with brain pathology standard T1 and T2 sequences were applied. No contrast agent was administered. T2* Gradient Echo-Planar Imaging (FFE-EPI: TR, 1,500 ms; TE, 27 ms; flip angle, 60°; matrix, 80 × 80; slice thickness, 3 mm with 0-mm gap, 210 volumes in series; TA 5 min 15 s; FOV, 240 mm × 240 mm), and 3D high-resolution T1 sequence (T1-TFE: TR, 7.44 ms; TE, 3.6 ms; slice thickness, 1 mm; matrix, 260 × 240; FOV, 260 mm × 240 mm) were applied for functional imaging and anatomical reference, respectively. During the resting state, acquisition subjects were asked to consciously attend to the fixation point presented in the center of the visual field and not to think of anything specific. The fixation point was presented *via* MRI-compatible goggles (NNL fMRI VisualSystem).

### Statistical Analyses

Data analyses were performed using the SPM12 toolbox (Wellcome Department of Imaging Neuroscience, London, UK, www.fil.ion.ucl.ac.uk/spm) implemented in MATLAB (Mathworks Inc., Sherborn, MA, USA). Single-subject data were pre-processed with Data Processing Assistant for Resting State fMRI (DPARSF v2.3, Chao-Gan & Yu-Feng 2010). The first five fMRI volumes were discarded to allow the BOLD signal to reach steady state. Functional scans were corrected for slice timing, realigned to the first image of the time series, and normalized at 3 mm × 3 mm × 3 mm in reference to a standard brain atlas (SPM12 MNI space). Participants with movement exceeding a 2-mm vector of translation were excluded from further analyses (eight subjects met the criteria—2 form CON group, 2 from FD group, 3 from IBD group, and 1 from IBS group). Subsequently the T1 anatomical images were segmented and the signal from the white matter and cerebrospinal fluid was extracted. Resting state data were further denoised using head motion scrubbing regressors and additional nuisance regressors that included white matter and cerebrospinal fluid signals. Finally, the data were low-pass filtered (with 0.1 Hz cut-off) and smoothed with 4-mm full width at half maximum Gaussian kernel. The purpose of the pre-processing was to remove various kinds of artifacts (i.e. the physiological noise), and to condition the data, to maximize the sensitivity of later statistical analyses, and to increase statistical validity.

In group resting state data analysis, the Group ICA of fMRI Toolbox [GIFT v4.0, icatb.sourceforge.net] (32) and a natural gradient (infomax) algorithm were used. The number of independent components was set at 20 to avoid anterior-posterior split of the Default Mode Network. Also, an ICASSO spell method was introduced (with ten times repeating of the ICA analysis) to increase the validity of the analysis. After subject-wise data concatenations, ICA was performed for all groups (CON, FD, IBD, IBS) in three stages:

- principal component analysis (PCA), which reduced each of the subject's fMRI data to predefined number of components;
- ICA algorithm (Infomax) application;
- back reconstruction for each individual subject's data, resulting in time courses and spatial maps of components.

For each of the groups 20 components were resembled. The Default Mode Network was identified with the use of spatial matching to the GIFT's binary DMN template. All independent components were converted to z-maps (32, 33). Each z-score represented the fit of a specific voxel BOLD time course to the time course of the group averaged component. The z-maps of the reconstructed subjects' DMN networks were further compared in SPM12 second-level one-way ANOVA with age as an additional covariate. Each of the group of interest (FD, IBS, IBD) DMN was contrasted with the control group using two sample t-test. Additionally, some joint intergroup comparisons including “the gastroenterological diseases vs control” (all 3 groups—FD, IBS, IBD) and “functional gastroenterological diseases vs control” (2 groups—FD and IBS) were performed with F-test. The results were all masked with the GIFTS's binary DMN template. Due to our *a priori* hypothesis whole-brain analysis was restricted to the DMN only (defined by the GIFT's template). For that purpose, a small volume correction was applied on cluster level. Clusters were regarded as significant when falling below an initial uncorrected voxel threshold of 0.001 and a topological False Discovery Rate corrected threshold of.05 adjusted for the small volume.

## Results

**Figure 1:**
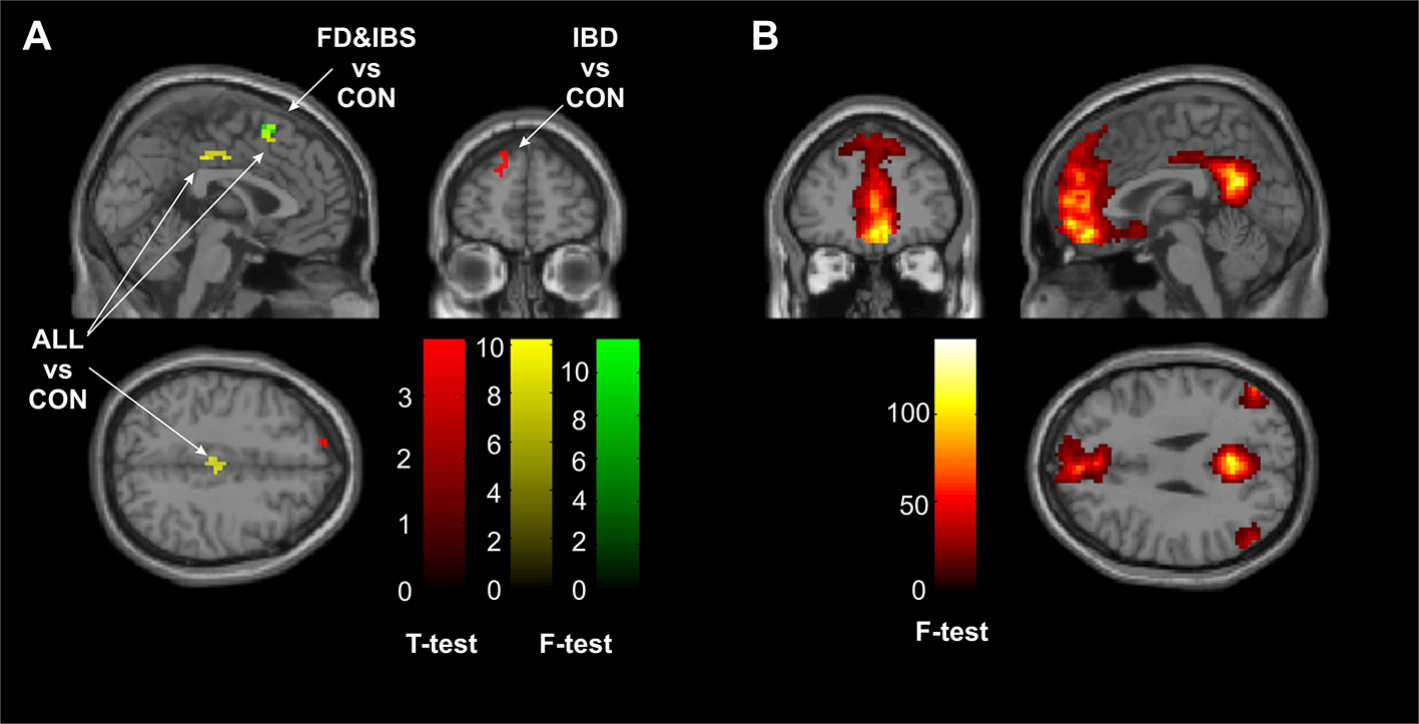
**(A)** Significant (cluster corrected p_FDR_ < .05) clusters of intergroup comparisons in contrast with the control group. The red cluster defines area of additional DMN connectivity of the IBD group, the green cluster represents a region of auxiliary connectivity of the DMN shared by both FD and IBS groups, whereas the yellow clusters underpin regions of increased DMN connectivity shared by all of the gastrointestinal diseases. **(B)** A map of the main effect of the Default Mode Network as reconstructed during the Independent Component Analysis across all of the subjects—patients and controls combined (p_FWE_ < 0.05). CON, the control group; FD, the functional dyspepsia group; IBS, the irritable bowel syndrome group; IBD, the inflammatory bowel disease group; ALL, all diseases (FD, IBS, and IBD).

The automated matching of the Default Mode Network resulted in a component replicable amongst groups as shown in **Figure 1B**. No anterior-posterior split of the DMN was observed.

### DMN of Given Diseases

The two-sample t-test comparisons revealed significant effect only when contrasting the IBD group with the CON group. The IBD group showed auxiliary area of DMN connectivity in one cluster in the superior frontal gyrus of the left hemisphere (peak coordinates = −18, 57, 30, Z = 3.64, k = 28, corrected cluster p_FDR_ = 0.030). The cluster is visualized in red in **Figure 1A** (the “IBD vs CON”).

### Joint Group Comparisons

When comparing all the patients and the controls, two clusters reached significance. First one located in the left cingulum (peak coordinates = 3, −15, 39; Z = 4.17; k = 23; corrected cluster p_FDR_ = 0.004), second one located in the left supplementary motor area (peak coordinates 0 15 54, Z=3.77, k=14, corrected cluster p_FDR_=0.021). In both cases the DMN of the patients showed increased connectivity in those areas. The clusters are visualized in yellow in **Figure 1A** (the “ALL vs CON”).

When restricting the comparison to functional gastroenterological diseases only—one cluster reached significance. It was located in left supplementary motor area (peak coordinates = −3, 12, 57; Z = 3.87; k = 14; cluster corrected p_FDR_ = 0.043) and roughly overlaps with one of the clusters of “the gastroenterological diseases vs control” reported in previous paragraph. The direction of the alternation remained unchanged (the DMN of the functional gastrointestinal patients was increased in the area as compared with the controls). The cluster is visualized in green in **Figure 1A** (the “FD and IBS vs CON”).

All results are summarized in **Table 2**.

**Table 2:** Summary results of the intergroup comparisons.

		X	y	z	No. of voxels	T-test/F-test	Z	Cluster corrected p_FDR_
IBD vs CON	LH superior frontal gyrus	−18	57	30	28	3.86	3.64	.030
ALL vs CON	LH cingulum	3	−15	39	23	10.18	4.17	.004
	LH supplementary motor area	0	15	54	14	8.51	3.77	.021
FD and IBS vs CON	LH supplementary motor area	−3	12	57	14	11.54	3.87	.043

Anatomical labels according to Automated Anatomical Labeling tool. All reported clusters are significant at cluster p < .05 threshold with topological False Discovery Rate small volume correction.

CON, the control group; FD, the functional dyspepsia group; IBS, the irritable bowel syndrome group; IBD, the inflammatory bowel disease group; ALL, all diseases (FD, IBS and IBD); x, y, z, peak coordinates in MNI space; Z, Z-score.

## Discussion

Functional diseases of gastrointestinal tract, frequently encountered worldwide, have a negative influence on the quality of life and cause significant costs in health care systems. Diagnostic criteria are based on Rome III Criteria (5, 34). Functional dyspepsia is the most common of FIGDs, diagnosed in approx. 25% of population. In 15% to 24% of western world population irritable bowel syndrome is encountered. Functional diseases of gastrointestinal tract are more common in young women, in case of IBS 2 to 3 times in comparison to men (35).

Patients suffering from FIGDs more often present with emotional distress and lower stress tolerance. Patient's constitution and response to stress may influence biological processes within the central nervous system and through autonomic nervous system trigger somatic reactions from digestive tract, leading to decrease in quality of life (36). Brain-gut axis disorders are also taken into account in etiology of FIGDs symptoms, mainly with regard to lowering the pain threshold (37). Correlation between anxiety and lowering the pain threshold, epigastric discomfort, burning, and early satiation has been proven. Patients suffering from FIGDs are also prone to guilt, increased self-criticism, catastrophical thoughts, and focusing on failure, they are said to cope worse with everyday problems. However, no correlation between FIGDs and lifespan has been reported (38).

Since 1937 when Papez first described neural pathway in the brain thought to be involved in the cortical control of emotion, the limbic system has been under scrutiny. At first brain studies have been mainly based on observation of its response to damage or stimulation. New possibilities emerged with introduction and development of imaging techniques, such as single-photon emission computed tomography (SPECT), positron emission tomography (PET), and functional magnetic resonance imaging (fMRI). Ample research has been conducted in order to investigate neuroanatomy and recognize which areas of the brain activate while different emotional states are being induced in healthy individuals. Phan et al. (39) in their meta-analysis of 55 studies with the use of PET and fMRI in healthy subjects showed that fear is associated with increase of activity in amygdalae, while sadness with activation of the perigenual anterior cingulate cortex. Medial part of prefrontal cortex plays a significant role in evaluation and processing of emotional data.

Our results showed that DMNs in study and the control group differed in left superior frontal gyrus, left cingulum and in the left supplementary motor area. Number of studies focused on functioning of the brain in FGIDs (15, 40–42). The study of Liu at al. (2013) focused on orbitofrontal cortex (OFC), parietal cortex, pregenual anterior cingulate cortex (pACC), and dorsomedial prefrontal cortex (dmPFC) (43). There are several factors that may contribute to the discrepancies between our studies. Most important one is the study population. In our research we focused on the joint effect of the different FGIDs which may have covered different brain responses specific for the each of the group of patients separately. Also the image analysis proposed by Liu at al. did not include detrending and filtering of the data which may resulted in different SNR ratio (signal-to-noise ratio) and also affect the results. We interpret our results with reference to the model of the brain-gut axis as explained by Mayer (2005) (44). The author describes the homeostatic afferent processing network as the one responsible for processing visceral and somatic stimuli (both painful and non-painful). What is most important in the model, is a broad understanding of homeostasis which includes responding to emotional stimuli as well. This point of view seems to be crucial for FGIDs patients description. Discovered differences in the DMNs can be interpreted as altered processing of homeostatic stimuli.

Despite that the Default Mode Network in gastric diseases is not yet well examined, there are multiple research explaining role of brain regions, which could give ideas of psychological functioning of this group of patients. Results of our study showed differences in three regions i.e. superior frontal gyrus of the left hemisphere, left cingulum, left supplementary motor area (see **Figure 1**) between particular groups. All diseases groups differed from control group in Default Mode Network pattern in left cingulum. Functions of cingulum are widely described, as a part of a limbic system it was always connected with emotions (including social interactions), motivation, and cognitive processes, such as memory and executive functions (45). However if the greater emphasis will be put on particular parts of cingulum, then its frontal connections are more likely consider as a attenuating in cognitive control, attention processes as well as pain, motor mechanisms, and reward signaling (46). Looking at cingulum in aspects of gastric diseases the role in amplifying emotional responses to pain signals described by Cohen et al. (47) seems to be the most important. As mentioned above, patients with gastric diseases often have lower stress tolerance and higher anxiety level. Thus different Default Mode Network pattern in cingulum region might be linked with often pain presence in examined gastric diseases.

Different DMN activation of superior frontal gyrus (SFG) was found in IBD group comparing to control. Functional brain imaging studies focused on chronic pain emphasis that chronic pain conditions involve mostly medial prefrontal cortical areas as well as subcortical limbic regions. Apkarian et al. (48) suggest that in majority of chronic pain diseases a shift away from brain areas engaged in sensory processing of pain to regions involved in emotional and motivational subjective states is observed. This observation would explain differences in DMN in SFG between IBD individuals and control group. Activation of these limbic/emotional structures related to subjective pain in IBD might result in psychological symptoms such as anxiety or dissatisfaction.

Last DMN with statistically relevant differences in region of supplementary motor area was noticed in comparison of functional gastric diseases (functional dyspepsia group, irritable bowel syndrome) and control groups. This brain area is considered to play role in facilitating spontaneous motor responses to auditory stimuli, and in supporting a flexible engagement of sensorimotor processes to enable auditory perception and imaginary (49). Reflecting these findings to our results may explain why symptoms of FD and IBS can appear as a react to particular sound or image.

The psychological description of the patients corresponds with this explanation. Patients are characterized as anxious and depressive; also, the comorbidity of the FGIDs and the psychiatric disorders regard mostly the mood and anxiety disorders (50), thus suggesting altered emotional processing as well.

In conclusion, it needs to be stressed that our study group involved patients suffering from both functional and non-specific inflammatory bowel diseases. Nevertheless, with a spectrum of changes in the study group (superior frontal gyrus of the left hemisphere, in the left cingulum and in the left supplementary motor area), we were able to find common features, differentiating the whole study group from the healthy controls.

## Data Availability Statement

The datasets generated for this study are available on request to the corresponding author.

## Ethics Statement

The studies involving human participants were reviewed and approved by The Bioethical Committee of The Military Medical Council. The patients/participants provided their written informed consent to participate in this study.

## Author Contributions

KS: planning and conducting the study, collecting data, drafting the manuscript. GP: collecting the patients and conducting the study. PN and AS: analysis and interpretation of data. KM: drafting the article it critically for important intellectual content. GR: conception and design. ES: final approval of the version to be published.

## Conflict of Interest

The authors declare that the research was conducted in the absence of any commercial or financial relationships that could be construed as a potential conflict of interest.
